# Association of the children's dietary inflammatory index (c-DII) with thiol/disulfide homeostasis in pediatric glycogen storage disease: a cross-sectional exploratory study

**DOI:** 10.3389/fnut.2026.1863028

**Published:** 2026-09-01

**Authors:** Burcu Kumru Akin, Emine Goksoy, Mehmet Keskin, Hakim Çelik

**Affiliations:** 1 Division of Nutrition and Diet, Gaziantep Cengiz Gökçek Maternity and Children's Hospital, Gaziantep, Türkiye; 2 Division of Pediatric Metabolism, Department of Pediatrics, Gaziantep Cengiz Gökçek Maternity and Children's Hospital, Gaziantep, Türkiye; 3 Department of Pediatric, Division of Pediatric Endocrinology and Metabolism, Gaziantep University Faculty of Medicine, Gaziantep, Türkiye; 4 Department of Physiology, Faculty of Medicine, Harran University, Şanlıurfa, Türkiye

**Keywords:** dietary inflammatory index, glycogen storage disease, medical nutrition therapy, oxidative stress, thiol/disulfide homeostasis

## Abstract

**Objective:**

The dietary pro-inflammatory burden observed among patients receiving medical nutritional therapies for glycogen storage disease (GSD) and its association with cellular redox balance have not been sufficiently elucidated in the literature. The aim of this cross-sectional study was to evaluate the association of the dietary inflammatory potential (Children's Dietary Inflammatory Index, c-DII) with systemic thiol/disulfide homeostasis and selected disease-specific clinical markers in pediatric patients with GSDI and GSDIII.

**Materials and methods:**

This cross-sectional study included 16 pediatric patients with GSD (GSDI, *n* = 8; GSDIII, *n* = 8) and 15 age-and sex-matched healthy controls. The inflammatory potential of the diet was calculated using 22 parameters obtained from 3-day retrospective dietary intake records and energy-standardized (1,000 kcal) c-DII. Serum dynamic thiol/disulfide homeostasis parameters were analyzed using a spectrophotometric method, and associations between c-DII scores and biochemical parameters were evaluated using correlation analyses.

**Results:**

The mean c-DII score in the GSD cohort was positive, indicating an overall pro-inflammatory potential of the observed dietary intake. In biochemical analyses, while native and total thiol levels in the GSD group were statistically significantly lower than in healthy controls (*p* < 0.05), the disulfide/native thiol and disulfide/total thiol ratios, indicating a cellular pro-oxidant shift, were significantly increased (*p* < 0.05). Elevated c-DII scores were found to be significantly associated with high disulfide levels and disulfide-based ratios. Furthermore, significant positive correlations were found between the c-DII score and subtype-specific clinical markers of damage (uric acid in GSDI, creatine kinase in GSDIII).

**Conclusion:**

Medical nutritional therapies, which are of vital importance in the management of GSD, may paradoxically be associated with a pro-inflammatory dietary burden and systemic antioxidant depletion. These preliminary findings suggest that integrating the energy-adjusted c-DII into clinical monitoring may offer an innovative strategy for assessing diet-related cellular stress in pediatric GSD. However, its definitive clinical utility requires confirmation in larger prospective studies.

## Introduction

Glycogen storage diseases (GSD) are inherited metabolic disorders characterized by chronic metabolic stress and organ-specific complications (hepatomegaly, hypoglycemia, myopathy, etc.), resulting from enzyme deficiencies in glycogen synthesis or breakdown. GSDI is caused by defects in the glucose-6-phosphatase complex and is typically associated with fasting intolerance, severe hypoglycemia, hyperlactatemia, hyperuricemia, and hepatomegaly; in contrast, GSDIII arises from a deficiency in glycogen debranching enzymes and presents with varying degrees of hepatic, muscular, and occasionally cardiac involvement. The primary approach to reducing complications and maintaining growth and development in patients with GSDI and GSDIII is lifelong medical nutritional therapy aimed at preventing hypoglycemia (1, 2). However, the cellular burden created by the disease's inherent genetic and metabolic nature renders the body extremely vulnerable to systemic inflammation and secondary oxidative stress (3).

In addition to this metabolic vulnerability, the literature has not sufficiently clarified whether the medically supervised dietary treatments mandated for GSD management may be associated with diet-related cellular risk. In GSDI, the high load of uncooked cornstarch (UCCS) accompanying fructose and galactose restriction; and in GSDIII, high-protein/high-fat dietary strategies implemented to suppress myopathy, result in patients having a diet limited in natural antioxidants such as fruits and vegetables (4–6). Treatment strategies for GSD patients may expose them to a diet-induced pro-inflammatory metabolic cycle and cellular stress (7, 8). The Dietary Inflammatory Index for Children (c-DII), developed in recent years to measure this inflammatory and oxidative capacity of the diet, has emerged as a powerful tool for predicting systemic inflammation in various chronic diseases (9, 10). This index is based on scoring specific nutrients according to their effects on pro-inflammatory cytokines such as CRP, IL-6, and TNF-α (positive coefficients) or their supportive effects on anti-inflammatory markers such as IL-4 and IL-10 (negative coefficients). A high c-DII score indicates a pro-inflammatory diet, while low (negative) scores reflect an anti-inflammatory pattern (9, 11). Nevertheless, data on the cellular implications of this dietary inflammatory burden in pediatric inborn errors of metabolism (IEM) receiving medical nutrition therapy are quite limited in the literature.

Detecting potential diet-related redox imbalances at an early stage is critical for preventing organ damage. Conventional oxidative stress markers total oxidant level (TOS) and total antioxidant level (TAS) may fail to accurately reflect cellular redox balance, particularly in specific metabolic conditions such as the hyperuricemia inherent in GSDI, as they can yield false-positive elevations (12, 13). Thiols constitute a fundamental component of the antioxidant defense system, and their reversible oxidation to disulfide bonds reflects changes in the cellular redox environment. A shift in this balance toward disulfide formation indicates a pro-oxidant state and has been associated with inflammation, metabolic stress, and tissue damage in various clinical conditions. The dynamic thiol/disulfide balance is an innovative biomarker capable of detecting early-stage cellular damage in a manner that is far more sensitive and specific than conventional tests (14, 15).

To the best of our knowledge, there are no studies in the literature examining the relationship between dietary pro-inflammatory burden (c-DII) and the dynamic thiol/disulfide balance at the cellular level in patients with GSD who are receiving specific medical nutritional therapy. Based on this gap, the aim of our study is to investigate the association between the inflammatory load of the diet and systemic redox balance in pediatric GSDI and GSDIII patients and to examine whether c-DII is associated with subtype-specific biochemical markers, including uric acid in GSDI and creatine kinase in GSDIII, in addition to thiol/disulfide homeostasis parameters. The hypothesis is that patients with GSD will have an impaired thiol/disulfide balance compared to healthy controls, and that higher c-DII scores will be associated with a shift toward a more pro-oxidant redox profile.

## Materials and methods

### Study design and participants

This study is a single-center, cross-sectional study conducted at the Pediatric Metabolism Clinic of the Gaziantep Cengiz Gökçek Maternity and Children's Hospital. The study included patients with GSDI (*n* = 8) and GSDIII (*n* = 8) who were under regular medical nutritional therapy, as well as age- and sex-matched healthy controls (*n* = 15). Healthy controls were recruited from children attending the same hospital for routine outpatient evaluation who had no known chronic, metabolic, inflammatory, or acute infectious conditions. Eligibility was assessed through medical history and clinical evaluation, and controls were frequency-matched to the patient group by age and sex. The inclusion criteria were: age between 2 and 18 years, a genetically confirmed diagnosis of GSDI or GSDIII, regular follow-up at the metabolic diseases clinic for at least 12 months prior to enrollment, receiving stable medical nutrition therapy for at least 12 months, and being metabolically stable at the time of sampling. Metabolic stability was defined as the absence of acute metabolic decompensation, infection, or hospitalization within the previous 3 months. The study protocol was conducted in accordance with the principles of the Declaration of Helsinki and was approved by the Ethical Committee of Gaziantep University Faculty of Medicine (Decision No. 2022/449, Date: April 12, 2023). Written informed consent was obtained from the parents or legal guardians of all children included in the study.

All patients were regularly followed by a multidisciplinary pediatric metabolic disease team, and their medical nutrition therapy was planned and monitored by a dietitian experienced in inherited metabolic disorders. In GSDI, dietary management included frequent carbohydrate provision, uncooked cornstarch and/or modified uncooked cornstarch, and restriction of fructose and galactose. In GSDIII, management included avoidance of prolonged fasting, uncooked cornstarch when clinically indicated, and relatively higher protein intake. Pharmacological treatments were individualized according to GSD subtype, clinical status, and patient-specific complications as part of routine clinical care. During routine follow-up, dietary counseling was individualized according to age, clinical condition, biochemical findings, growth, and food tolerance, with efforts to improve dietary quality within the limits of the prescribed metabolic restrictions.

### Anthropometric measurements and dietary analysis

Anthropometric measurements (body weight and height) were taken for all participants at the time of their clinic visit, in accordance with standard protocols, while they were wearing light clothing and without shoes. The data obtained were analyzed using the WHO Antro and Antro Plus programs. Age-and sex-specific standard deviation scores (Z-scores) for weight and height were calculated.

The 3-day retrospective dietary intakes of the GSD patients included in the study were collected in person by a dietitian specializing in metabolic disorders. To reduce potential recall and portion-size estimation errors, food amounts were assessed using standard household measures and/or kitchen scales, and photographic documentation provided by the participants was reviewed for portion-size verification when available. Detailed dietary intake data were collected only from patients with GSD because the original dietary assessment was designed to examine the relationship between dietary inflammatory potential and redox parameters within the medically treated GSD cohort. Therefore, c-DII scores were not available for the healthy control group. All dietary data were analyzed in detail using the Nutrition Information System (BeBIS 8.2) software. The BeBIS database incorporates food composition data relevant to foods commonly consumed in Türkiye, drawing on national and established international food composition sources, and provides the macro- and micronutrient values required for the present dietary analyses. Macronutrients (carbohydrates, protein, total fat), fiber, cholesterol, saturated fatty acids, monounsaturated and polyunsaturated fatty acids, vitamins (vitamin A, vitamin C, vitamin D, vitamin E, vitamin B6, vitamin B12, folate, thiamine, riboflavin, niacin), minerals (iron, magnesium, zinc) and beta-carotene were calculated separately for each child. These 22 dietary parameters were then entered into the energy-adjusted c-DII algorithm.

In this study, the inflammatory potential of the patients' diets was assessed using the “Children's Dietary Inflammatory Index (c-DII)” algorithm-validated for children by Khan et al. (9)-which is based on the original DII developed by Shivappa et al. (11). In this context, of the 45 food parameters included in the original DII formulation, the 22 food parameters obtained from the dietary intake records in our study were included in the analysis. To eliminate the confounding effect that could arise from high inter-individual variations in total energy intake among children in the growth and development phase, the density method, in which energy is controlled was applied in the c-DII calculation (16, 17). According to this method, the dietary intake data obtained from the participants and the mean and standard deviation values from the global child reference dataset used for comparison were standardized by converting them to amounts per 1,000 kcal per day (9, 16). These individual intake values, calculated per 1,000 kcal, were compared with the global reference values, also based on 1,000 kcal, and converted into Z-scores (11). The obtained Z-scores were converted to percentile values based on the standard normal distribution to minimize the effect of outliers in the data and were centered between −1 and +1 to ensure symmetry (11). In the final step, these centered scores obtained for each dietary parameter were multiplied by fixed inflammatory effect coefficients derived from the literature that reflect the inflammatory capacity of the respective nutrient, summed, and used to calculate each child's c-DII score (9, 11).

To categorize the dietary inflammatory burden, the study cohort was stratified into two groups based on the median c-DII score (1.8) as the cut-off point: ‘Low Inflammatory Risk' (c-DII < 1.8) and ‘High Inflammatory Risk' (c-DII ≥ 1.8). These categories do not represent externally validated or clinically established risk thresholds.

### Biochemical analyses

#### Blood sample collection

Blood samples for routine laboratory tests, in addition to oxidative stress parameters, were collected during routine outpatient follow-up visits when patients were clinically stable and showed no symptoms of acute decompensation or infection.

Venous blood samples (5 ml) were collected into sterile serum separator tubes, allowed to clot at room temperature for 30 min, and then centrifuged at 1,500 × g for 10 min. The separated serum samples were stored at −86 °C until the day of analysis. On the day of biochemical analysis, serum samples were slowly thawed on ice to preserve cellular redox integrity and homogenized immediately prior to analysis.

#### Thiol/disulfide homeostasis and measurement of oxidative stress parameters

Serum dynamic thiol/disulfide homeostasis parameters were analyzed at the Laboratory of the Department of Physiology, Faculty of Medicine, Harran University, using a spectrophotometric method based on the Erel and Neşelioğlu method (14) as previously described in the literature, using a Varioskan LUX multimode microplate reader (Thermo Fisher Scientific, Waltham, MA, USA). Systemic redox balance was assessed based on the obtained native thiol and total thiol levels by calculating the ratios of disulfide/native thiol, disulfide/total thiol, and native thiol/total thiol (%).

Serum TOS and TAS levels were measured using commercial kits (Rel Assay Diagnostics, Gaziantep, Turkey) based on colorimetric methods previously developed by Erel (18). The oxidative stress index (OSI) was calculated using the standard formula found in the literature [OSI = (TOS / TAS) × 100].

### Statistical analysis

SPSS software (v18.0, SPSS Inc., Chicago, IL, USA) was used for the statistical analysis of the data. The distribution of continuous variables was assessed using the Shapiro–Wilk test, histograms, and skewness–kurtosis values. Descriptive statistics were presented as means ± standard deviation (SD) for normally distributed continuous variables and as median (Min–Max) for non-normally distributed continuous variables. Categorical variables were presented as number and percentage [*n* (%)], and group comparisons were performed using Fisher's exact test. To compare continuous variables between two independent groups (GSD patients and healthy controls), the Independent Samples *t*-test was used for normally distributed parameters, and the Mann–Whitney *U*-test was used for non-normally distributed parameters. For more than two groups, one-way ANOVA (parametric) or the Kruskal–Wallis test (non-parametric) was used, with *post-hoc* analyses performed as needed. Given the limited sample size and the potential influence of individual observations, associations between c-DII scores and biochemical parameters were examined using Spearman's rank correlation coefficient. Statistical significance was defined as a *p*-value < 0.05. A *post hoc* power analysis based on the primary comparison of serum native thiol levels between the overall GSD group and healthy controls showed a large observed effect size (Cohen's *d* = 1.29) and an estimated statistical power of 93.5% (1–β = 0.935) at a two-sided α level of 0.05. This estimate applies only to the primary overall comparison and not to the subtype, median-based c-DII, or correlation analyses, which were considered exploratory because of their limited sample sizes.

## Results

The study included 16 patients with GSD (GSDI [*n* = 8] and GSDIII [*n* = 8]) followed at the Pediatric Metabolism Clinic of Gaziantep Cengiz Gökçek Hospital of Obstetrics and Pediatrics, as well as a healthy control group (*n* = 15). When the demographic characteristics of the groups were examined, it was observed that the ages of the GSD patients and the control group were similar, and there was no statistically significant difference between them (*p* > 0.05). When anthropometric parameters were evaluated, it was determined that body weight-for-age and height-for-age Z-scores were statistically significantly lower in all GSD patients and in the GSD subgroups compared to healthy controls (*p* < 0.05). In particular, this marked delay in height growth was found to be even more pronounced in the GSDI group. The age and sex distributions were similar between the study groups, with no statistically significant differences (*p* > 0.05). The demographic characteristics and anthropometric measurements of the study and control groups are presented in Table 1.

**Table 1:** Comparison of demographic characteristics and anthropometric measurements between groups.

Parameters	Control (*n* = 15)	GSD (*n* = 16)	*p* ^1^	GSDI (*n* = 8)	GSDIII (*n* = 8)	*p* ^2^
Age (years)	7.37 ± 5.88	7.19 ± 4.28	0.924	7.57 ± 5.22	6.81 ± 3.42	0.954
**Sex**, ***n*** **(%)**			1.000			0.727
Female	8 (53.3)	8 (50.0)		5 (62.5)	3 (37.5)	
Male	7 (46.7)	8 (50.0)		3 (37.5)	5 (62.5)	
Weight-for-age Z score	0.48 ± 1.28	−1.11 ± 0.87	**0.032**	−1.25 ± 0.46^a^	−1.13 ± 1.21^a^	**0.023**
Height-for-age Z score	0.33 ± 1.10	−2.43 ± 0.42	**0.025**	−2.75 ± 1.33^b^	−2.12 ± 1.63^b^	**0.015**

*p*^1^Two independent groups (Control and GSD patients) Independent Samples *t*-test, *p*^2^Three groups (Control, GSDI and GSDIII) One-Way ANOVA, *post-hoc* Tukey HSD test for continuous variables and Fisher exact test for sex distribution, *p* < 0.05 Mean ± standard deviation.

^a^Weight-for-age Z score was significantly lower in patients with GSDI (*p* = 0.003) and GSDIII (*p* = 0.023) compared to the control group.

^b^Height-for-age Z score was significantly lower in patients with GSDI (*p* = 0.002) and GSDIII (*p* = 0.012) compared to the control group.

When the thiol/disulfide balance in the study groups was examined, it was found that native thiol and total thiol levels were statistically significantly lower in all GSD patients compared to healthy controls (*p* < 0.05). *Post-hoc* subgroup analyses demonstrated that the decrease in thiol levels was statistically significant in both GSDI and GSDIII patients compared to the control group; reduced antioxidant defense was observed in both patient groups. When disulfide ratios were examined, it was found that the disulfide/native thiol and disulfide/total thiol ratios were statistically significantly higher in all GSD patients compared to healthy controls (*p* < 0.05). Consistent with this increase in disulfide levels, the native thiol/total thiol ratio was also found to be significantly lower in GSD patients compared to the control group (*p* < 0.05). In the *post-hoc* analysis, it was observed that the disulfide/native thiol and disulfide/total thiol ratios were significantly higher in GSDIII patients compared to healthy controls, while the native thiol/total thiol ratio was significantly lower (*p* < 0.05). When examining markers of oxidative stress, TAS levels were found to be statistically significantly higher in GSD patients compared to controls (*p* < 0.05). *Post-hoc* analyses revealed that this elevation was specifically attributable to the GSD type 1 group. TAS levels in GSDI patients were significantly higher than those in healthy controls (*p* < 0.05).

When evaluated in terms of c-DII scores, no significant difference was observed between the c-DII scores of patients with GSD type 1 and type 3 (*p* > 0.05). All biochemical, redox balance, and nutritional data are summarized in Table 2.

**Table 2:** Comparison of dietary inflammatory index and biochemical parameters between groups.

Parameters	Control (*n* = 15)	GSD (*n* = 16)	*p* ^1^	GSDI (*n* = 8)	GSDIII (*n* = 8)	*p* ^2^
Thiol/Disulfide Homeostasis						
Native thiol (μmol/L)	737.54 ± 190.05	529.07 ± 129.25	**0.001**	544.71 ± 150.12^a^	513.43 ± 112.70^a^	**0.005**
Total thiol (μmol/L)	841.35 ± 188.62	712.44 ± 128.19	**0.037**	699.42 ± 122.74	725.47 ± 140.57	0.102
Disulfide (μmol/L)	54.97 ± 46.09	82.51 ± 35.05	0.074	77.34 ± 37.55	87.67 ± 34.08	0.176
Disulfide/native thiol (%)	8.28 ± 7.78	17.57 ± 10.88	**0.011**	16.03 ± 7.78	19.10 ± 11.69^b^	**0.034**
Disulfide/total thiol (%)	7.08 ± 5.47	12.01 ± 5.55	**0.013**	11.37 ± 5.57	12.65 ± 5.82^c^	**0.044**
Native thiol/total thiol (%)	87.50 ± 8.01	74.07 ± 11.62	**0.001**	77.25 ± 11.14	70.89 ± 11.92^d^	**0.002**
Oxidative Stress Markers						
TAS (mmol Trolox Equiv./L)	0.72 ± 0.17	0.98 ± 0.31	**0.008**	1.07 ± 0.39^e^	0.89 ± 0.18	**0.012**
^*^TOS (μmol H2O2 Equiv./L)	21.90 (16.40–34.63)	25.31 (13.71–53.75)	0.247^*^	26.34 (18.10–33.75)	24.65 (13.71–53.75)	0.421^*^
OSI (Arbitrary Unit)	3.20 ± 0.62	3.01 ± 1.57	0.671	2.90 ± 1.74	3.12 ± 1.49	0.861
Dietary Inflammatory Index						
c-DII	–	0.55 ± 2.91	–	0.84 ± 3.41	0.27 ± 2.52	0.709^#^

*p*^1^Two independent groups (Control and GSD patients) Independent Samples *t*-test, *p* Three groups (Control, GSDI and GSDIII) One-Way ANOVA, *post-hoc* Tukey HSD test, ^#^*p*^2^Two independent groups (GSDI and GSDIII) Independent Samples *t*-test Mean ± standard deviation, *p* < 0.05.

^*^p^1^Two independent groups (Control and GSD patients) Mann–Whitney U-test, ^*^p^2^Three groups (Control, GSDI and GSDIII) Kruskal-Wallis test, median (minimum-maximum) *p* < 0.05.

^#^c-DII was calculated only for patients with GSD.

^a^Native thiol was significantly lower in patients with GSDI (*p* = 0.031) and GSDIII (*p* = 0.011) compared to the control group.

^b^Disulfide/native thiol was significantly higher in patients with GSDIII (*p* = 0.040) compared to the control group.

^c^Disulfide/total thiol was significantly higher in patients with GSDIII (*p* = 0.049) compared to the control group.

^d^Native thiol/total thiol was significantly lower in patients with GSDIII (*p*=0.002) compared to the control group.

^e^TAS was significantly higher in patients with GSDI (*p* = 0.009) compared to the control group.

Bold values indicate statistically significant p-values (*p* < 0.05).

**Figure 1:**
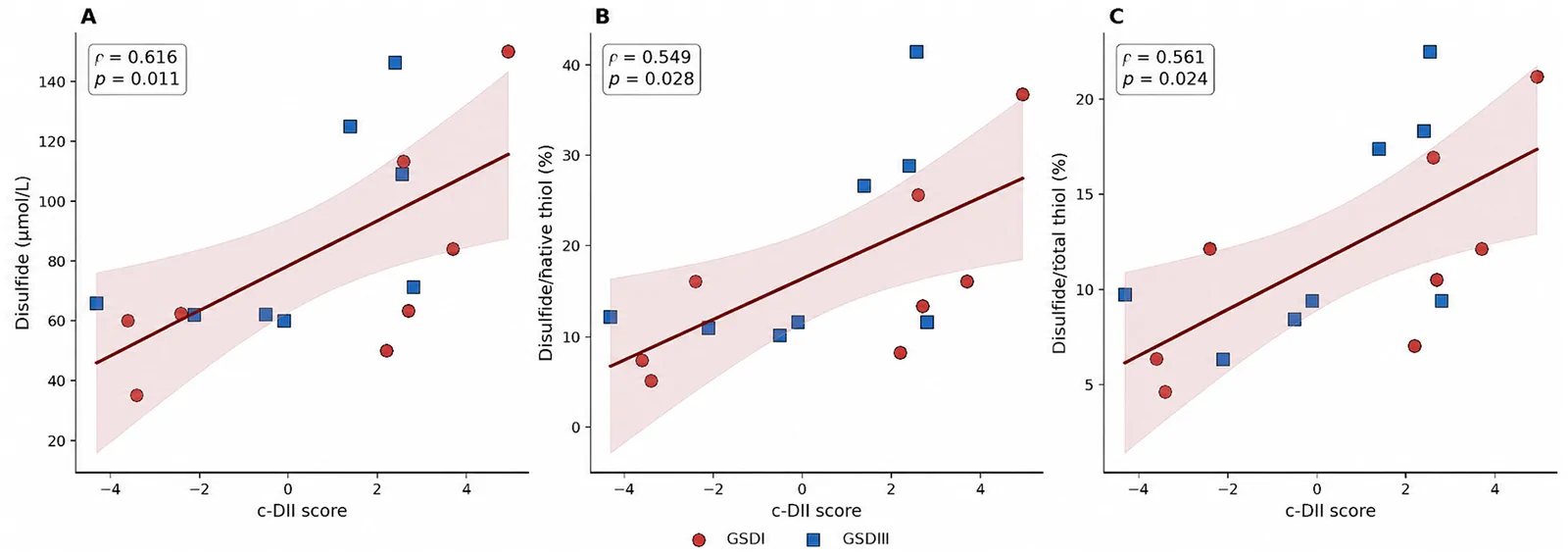
Correlations between c-DII score and thiol/disulfide homeostasis parameters in patients with glycogen storage disease Scatter plots showing the correlations between c-DII score and disulfide level, disulfide/native thiol ratio, and disulfide/total thiol ratio in the overall patient group. Symbols indicate GSD subtypes (GSDI and GSDIII), whereas the fitted regression lines and correlation coefficients represent analyses performed in all patients combined. Shaded areas indicate 95% confidence intervals. **(A)** Correlation between c-DII score and disulfide level (μmol/L). **(B)** Correlation between c-DII score and disulfide/native thiol ratio (%). **(C)** Correlation between c-DII score and disulfide/total thiol ratio (%) Spearman's rank correlation analysis; *p* < 0.05.

**Figure 2:**
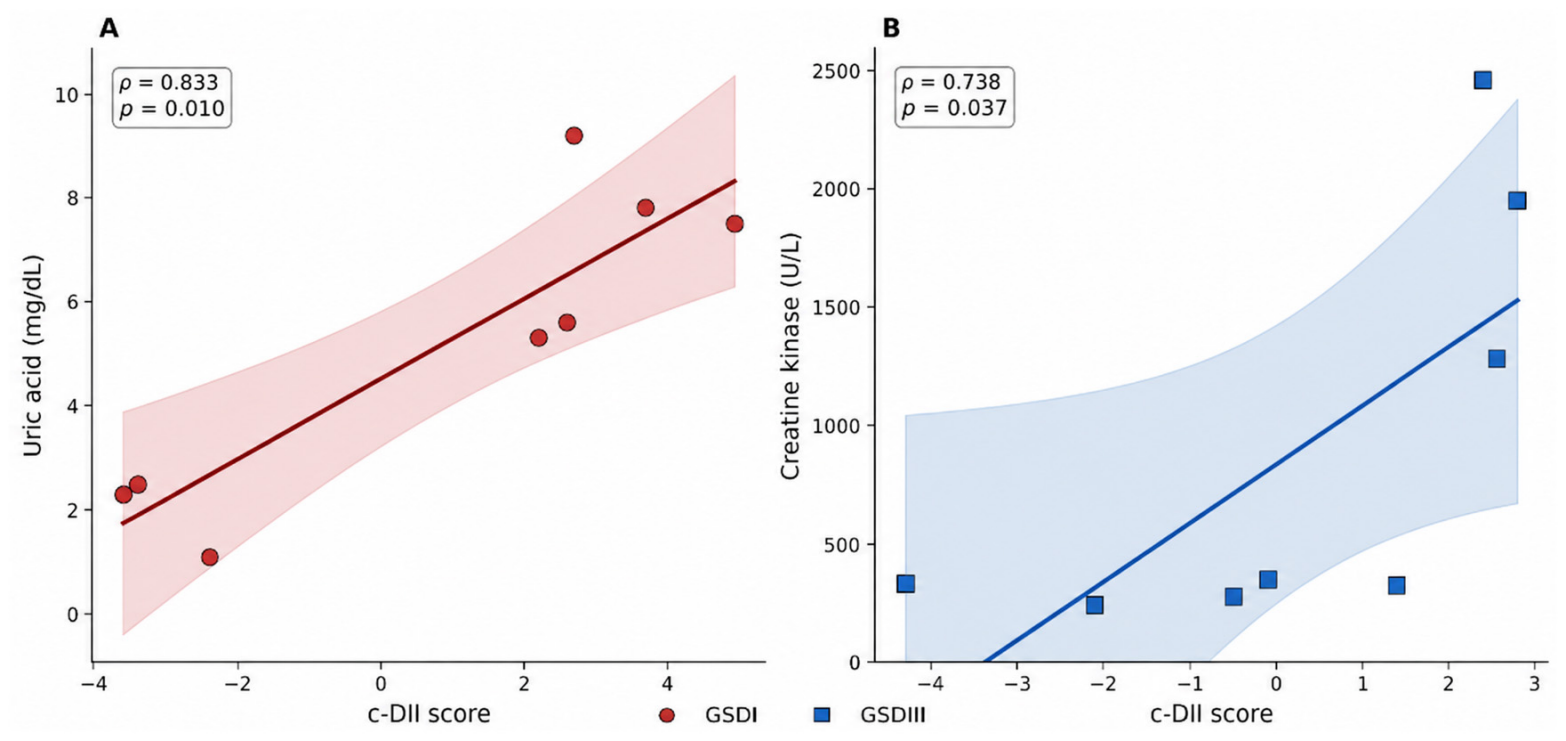
Correlations of c-DII score with uric acid and creatine kinase levels in patients with glycogen storage disease subtypes. Scatter plots showing the correlations between c-DII score and uric acid levels in patients with GSDI and creatine kinase levels in patients with GSDIII. Red circles represent patients with GSDI, and blue squares represent patients with GSDIII. Fitted regression lines and correlation coefficients are shown for each subgroup separately. Shaded areas indicate 95% confidence intervals. **(A)** Correlation between c-DII score and uric acid level (mg/dl) in patients with GSDI. **(B)** Correlation between c-DII score and creatine kinase level (U/L) in patients with GSDIII. Spearman's rank correlation analysis; *p* < 0.05.

The results of the correlation analyses showed that c-DII scores were significantly correlated with disulfide levels (ρ = 0.616, *p* = 0.011), the disulfide/native thiol ρ = 0.549, *p* = 0.028) and disulfide/total thiol (ρ = 0.561, *p* = 0.024) ratios (Figure 1). In the analyses performed, a statistically significant positive correlation was found between the c-DII score and uric acid levels (5.15 ± 2.94 mg/dl) in patients with GSDI (ρ = 0.833, *p* = 0.010). Similarly, a statistically significant positive correlation was found between the c-DII score and creatine kinase (CK) levels (905.50 ± 880.00 U/L) in patients with GSDIII (ρ = 0.738; *p* = 0.037) (Figure 2). Given the small number of participants in each subtype, these subgroup findings should be interpreted cautiously.

Patients were divided into two groups ‘Low Inflammatory Risk' (c-DII < 1.8) and ‘High Inflammatory Risk' (c-DII ≥ 1.8) using the median c-DII score of 1.8 as a study-specific exploratory cutoff (Table 3). In the analyses conducted, while no significant difference was observed in native and total thiol levels between the groups, it was found that disulfide levels were statistically significantly higher in the high-risk group compared to the low-risk group (*p* < 0.05). A borderline significant increase in the disulfide/total thiol ratio was observed in the high-risk group (*p* = 0.050). In addition, no statistically significant difference was found between the two risk groups in terms of TAS, TOS, and OSI values, which are classic markers of oxidative stress (*p* > 0.05) (Table 3). Given the small number of participants in each subgroup, these findings should be interpreted as preliminary exploratory patterns rather than definitive evidence of a clinically validated risk gradient.

**Table 3:** Thiol/disulfide homeostasis and oxidative stress markers according to dietary inflammatory risk groups in the GSD cohort.

Parameters	Low inflammatory risk (c-DII < 1.8) (*n* = 8)	High inflammatory risk (c-DII ≥ 1.8) (*n* = 8)	*p*
Thiol/disulfide homeostasis			
Native thiol (μmol/L)	556.06 (390.020–821.62)	493.21 (263.25–617.16)	0.208
Total thiol (μmol/L)	728.64 (515.46–978.74)	700.91 (485.50–802.86)	0.400
Disulfide (μmol/L)	62.00 (35.17–124.88)	98.45 (50.11–150.11)	**0.046**
Disulfide/native thiol (%)	11.23 (5.14–26.52)	20.80 (8.23–41.41)	0.066
Disulfide/total thiol (%)	8.89 (4.66–17.33)	14.53 (7.07–22.45)	**0.050**
Native thiol/total thiol (%)	80.94 (57.78–90.67)	70.94 (54.22–85.87)	0.172
Oxidative stress markers			
TAS (mmol Trolox Equiv./L)	0.83 (0.73–1.58)	1.07 (0.40–1.34)	0.528
TOS (μmol H2O2 Equiv./L)	25.31 (18.10–53.75)	24.45 (13.71–33.75)	0.100
OSI (Arbitrary Unit)	3.14 (1.15–6.05)	2.84 (1.32–6.80)	0.528

^*p*^Mann–Whitney U-test, median (minimum-maximum) *p* < 0.05. Bold values indicate statistically significant p-values (*p* < 0.05); *p* = 0.050 is shown in bold to indicate borderline statistical significance.

## Discussion

This exploratory study provides novel insights into the relationship between dietary inflammatory burden and systemic redox balance in pediatric patients with GSDI and GSDIII receiving long-term medical nutritional therapy. The primary finding is that, compared with healthy controls, the overall GSD group exhibited lower native and total thiol concentrations alongside higher disulfide-based ratios. Furthermore, within the GSD cohort, higher c-DII scores were significantly associated with a pro-oxidant shift (a less favorable thiol/disulfide profile). In subtype analyses, c-DII scores were positively correlated with uric acid levels in GSDI and creatine kinase levels in GSDIII. Although the cross-sectional observational design precludes causal inferences, these findings suggest that the higher dietary inflammatory potential of the observed intake may be associated with redox imbalance and subtype-specific biochemical markers in pediatric GSD.

Growth retardation observed in patients with GSD is a well-documented complication in the literature; it is primarily associated with the chronic metabolic stress caused by the disease, difficulties in glucose regulation, and the strict dietary modifications that must be implemented from an early stage to prevent hypoglycemia (such as the use of raw cornstarch and restrictions on fructose and galactose, etc.) (2, 19).

A review of the literature has shown that a high pro-inflammatory dietary load (DII/c-DII) increases systemic inflammation markers (CRP, IL-6, etc.) and triggers oxidative stress through classical pathways (20, 21). Similarly, it has been demonstrated in various clinical settings that dynamic thiol/disulfide homeostasis serves as a much more sensitive and innovative biomarker for reflecting cellular redox balance and early-stage oxidative damage compared to conventional markers (15, 22). Our study, by examining these two physiological pathways together, suggests that a high c-DII score may be associated not only with systemic inflammation but also with the accumulation of disulfides at the cellular level (pro-oxidant shift). Consequently, this diet-induced accumulation of disulfides can act as a critical triggering mechanism that exacerbates structural cellular stress in GSD pathogenesis by depleting thiol reserves, thereby paving the way for the development of clinical organ involvement ranging from progressive muscle wasting (myopathy/cardiomyopathy) to renal dysfunction (nephropathy/hyperuricemia) (23).

In our study, when the baseline redox status of patients with GSD was evaluated, it was found that native and total thiol reserves-which form the basis of cellular antioxidant defense-were statistically significantly depleted compared to healthy controls (*p* < 0.05). Parallel to this thiol depletion, the disulfide/native thiol and disulfide/total thiol ratios-indicators of cellular oxidation and pro-oxidant shift-were found to be statistically significantly elevated in the patient group (*p* < 0.05) (Table 2). This finding, which is consistent with the literature, demonstrates that the chronic metabolic stress inherent in GSD, the risk of recurrent hypoglycemia, and abnormalities in glucose regulation lead to continuous systemic production of reactive oxygen species (ROS), and that the thiol pool is depleted in an effort to neutralize these radicals (23–25).

In particular, when analyzing subgroup data by disease, this pro-oxidant shift in redox balance was statistically significantly more pronounced in GSDIII patients compared to healthy controls. This situation can be explained by the additional contribution of skeletal muscle involvement (myopathy)-a characteristic of the disease-and the intracellular oxidative burden caused by accumulated limit dextrin, in addition to liver involvement in GSDIII. As a result of the continuous breakdown of muscle tissue and the accumulation of limit dextrin, both systemic inflammation increases and oxidative stress deepens, rapidly converting the thiol pool into disulfide (3). Mitochondrial dynamics are adversely affected, particularly due to the physical damage caused by the accumulation of limit dextrin. As a result, energy production in mitochondria is disrupted, and they become more susceptible to oxidative damage (26). The fact that no significant difference in disulfide ratios was detected between GSDI and GSDIII groups indicates that, although both subtypes are associated with different organ-specific complications (hyperuricemia and myopathy), their ultimate cellular targets and the systemic redox damage they induce are of similar severity. This cellular thiol depletion we observed also shares biochemical parallels with other inborn errors of metabolism in the literature, which are characterized by the accumulation of toxic metabolites and chronic cellular stress. In a study conducted by Cam et al. (27) on inborn errors of metabolism of the intoxication type, the patients' native thiol/total thiol ratios were found to be significantly lower than those of the control group, while the levels of disulfide, disulfide/native thiol, and disulfide/total thiol were significantly higher in the patient group compared to the control group. These findings indicate that oxidative stress increases during metabolic episodes; they also demonstrate that poor metabolic control and non-compliance with treatment are directly associated with increased oxidative stress. In a thesis study that included patients with different types of GSD, it was found that total thiol levels in patients with GSDIII were significantly lower than in healthy controls, and that the thiol/disulfide balance was markedly disrupted. In addition, it was found that patients with poor metabolic control across all GSD disease types had significantly lower native thiol levels compared to those with well-controlled disease (28). In another study evaluating thiol/disulfide homeostasis in patients with maple syrup urine disease (MSUD), it was reported that thiol/disulfide balance remained intact in patients who adhered to medical nutritional therapy and were regularly monitored (29). Although the toxic agents that accumulate in various inborn errors of metabolism (e.g., phenylketonuria (PKU), MSUD, urea cycle defects) vary, the primary biochemical response of target tissues to this chronic metabolic and inflammatory stress is common (27, 29). In each condition, the rapid oxidation of native thiols—the organism's most critical early defense line—into disulfides and the depletion of the thiol pool emerge as a common pathophysiological pathway that accelerates cellular damage (14, 15).

In our study, when oxidative stress markers were evaluated, TAS levels were found to be statistically significantly higher in all GSD patients, and particularly in those with GSDI, compared to healthy controls. Although this finding initially gives the impression of increased antioxidant capacity, it is actually a reflection of hyperuricemia, one of the characteristic metabolic complications of GSDI. As emphasized in the literature, uric acid constitutes a large portion of the TAS value, and due to the pathologically elevated uric acid levels in GSDI patients, there appears to be an artificially high antioxidant capacity (13, 30, 31). Indeed, the positive correlation observed in our study between the c-DII score and uric acid levels (ρ = 0.833, *p* = 0.010) supports this finding. As the pro-inflammatory dietary load increases, the hyperuricemia profile in patients with GSDI worsens, and this situation leads to classical TAS measurements becoming “misleading” in reflecting cellular oxidation. To overcome this limitation, “thiol/disulfide homeostasis,” a much more sensitive and dynamic indicator of cellular oxidative stress, is being preferred as a new biomarker (15).

When the dietary origins of this pro-oxidant shift observed in cellular redox balance were examined, the mean c-DII score for the overall GSD cohort was found to be positive (0.55 ± 2.91). A positive mean c-DII value indicates that, on average, the observed dietary intake within this GSD cohort had an overall pro-inflammatory potential. In subgroup analyses, no statistically significant difference in c-DII scores was found between the GSDI (0.84 ± 3.41) and GSDIII (0.27 ± 2.52) groups (*p* > 0.05). This finding may reflect the composite nature of the c-DII rather than equivalent dietary composition or metabolic effects between the two subtypes. In addition to the continuous carbohydrate flow created by uncooked corn starch (UCCS) and/or modified UCCS-which are commonly used in both groups to prevent hypoglycemia-in GSDI, a high-carbohydrate diet is recommended due to the restriction of fruits and vegetables-which are natural sources of antioxidants-resulting from the restriction of fructose and galactose. In GSDIII, although there are no restrictions on fruits or vegetables (many of which are important sources of antioxidants), a high-fat, high-protein diet is recommended to prevent myopathy and support gluconeogenesis (1, 6). As emphasized in the literature, both high carbohydrate intake and high intake of animal protein, total fat, and saturated fat increase the diet's total inflammatory burden, thereby raising the score and potentially contributing to systemic inflammation (32). Therefore, nutritionally distinct dietary patterns may generate comparable overall c-DII scores through different combinations of dietary components. The c-DII may not fully distinguish whether the calculated inflammatory potential is predominantly driven by carbohydrate exposure, restricted antioxidant-rich foods, saturated fat, animal protein, or other dietary characteristics in this specialized population. Accordingly, the similar c-DII scores observed in GSDI and GSDIII should not be interpreted as evidence that the two medical nutrition strategies have identical inflammatory or metabolic effects. Therefore, although the dietary strategies applied in the two disease subtypes differ substantially, they may result in comparable calculated c-DII scores through different nutritional pathways. This similarity in total score should not be interpreted as evidence of an identical cellular risk profile. The positive mean c-DII observed in this medically treated GSD cohort may represent a dietary challenge that warrants attention when compared with findings reported in other IEM in the literature. Indeed, in the current literature, it has been reported that in conditions requiring a naturally protein-restricted diet, such as PKU, patients' DII scores are maintained at levels similar to those of healthy controls despite high carbohydrate and fat intake, thanks to the use of specific medical formulas enriched with micronutrients (33). However, the positive mean c-DII scores and reduced thiol reserves observed in GSD patients in our study suggest that the dietary and redox profiles of these patients may differ from those reported in some other IEM. Whether these differences are attributable to the prescribed therapy, actual dietary adherence, disease-related factors, or their interaction cannot be determined from the present data. An additional consideration is that c-DII scores were not available for the healthy control group because detailed dietary intake data were not collected from controls in the original study protocol. Consequently, the present study cannot determine whether the observed dietary inflammatory potential in the GSD cohort was higher than that of age- and sex-matched healthy children. It also cannot establish that medical nutrition therapy itself was the sole or primary source of the observed dietary inflammatory potential. Food preferences, adherence, family eating practices, cultural and socioeconomic factors, and the underlying metabolic disease may also have contributed. Future studies should include parallel dietary assessment in healthy controls and, where possible, comparison of prescribed diets with actual dietary intake.

The strong correlations we identified in our study between the c-DII score and markers of cellular oxidative stress (disulfide ratios) and organ damage (uric acid for GSDI and CK for GSDIII) may provide preliminary evidence of a possible association between dietary inflammatory potential and redox imbalance in GSD, rather than establishing a direct link with GSD pathophysiology. It is well established in studies of the general population and various chronic diseases that a high pro-inflammatory dietary load (DII) directly increases systemic oxidative stress (32, 34). In the context of GSD, where cellular defense mechanisms may already be vulnerable, our findings suggest that a higher dietary inflammatory potential may be associated with early-stage disulfide accumulation and lower thiol availability. Furthermore, the specific relationships between c-DII, uric acid, and CK suggest that the pro-inflammatory dietary load may be linked to disease-specific biochemical alterations. The literature emphasizes that anti-inflammatory dietary patterns are associated with reduced serum uric acid levels, and that elevated uric acid creates a positive feedback loop that exacerbates cellular oxidative damage by directly activating inflammatory pathways (e.g., NF-kB) (35). When the inherent predisposition to hyperuricemia in patients with GSDI is considered alongside the strong positive association identified in our study, higher c-DII scores may be associated with a less favorable uric acid and redox profile. However, given the small subgroup size and the unadjusted nature of the analysis, this finding should be regarded as exploratory and does not establish an independent or causal effect of dietary inflammatory potential. Furthermore, in cases of GSDIII characterized by myopathic involvement, the association observed between the c-DII score and CK levels—a key marker of muscle breakdown—is particularly noteworthy in this context. It is known in the literature that in GSDIII, the inability to fully break down the limit dextrin that accumulates in tissues leads to the release of ROS from damaged mitochondria and a depletion of antioxidant capacity, thereby creating a progressive damage process that continuously triggers cellular breakdown (increased CK levels) in skeletal muscle (3). The correlation between c-DII and CK observed in our study suggests that the additional oxidative burden associated with a more pro-inflammatory dietary pattern may contribute to the mitochondrial stress already present in muscle tissue. In other words, as the inflammatory potential of the diet increases, the cell's antioxidant (thiol) defense may become more vulnerable, and the myopathic breakdown process inherent in GSDIII may be reflected at a more pronounced biochemical level. Our study aims to contribute an innovative and integrative perspective to the literature by indicating that these two distinct pathophysiological mechanisms—hyperuricemia and metabolic dysfunction in GSDI, and myopathic breakdown in GSDIII—may converge, at least in part, in their association with a more pro-inflammatory dietary pattern.

One of the notable findings of our study is that, while disulfide levels in GSD patients as a whole did not differ statistically from those in healthy controls (Table 2); when patients were grouped based on the inflammatory burden of their diet (median c-DII ≥ 1.8), the increase in disulfide levels reached statistical significance (*p* < 0.05) in the high-risk group (Table 3). Furthermore, the disulfide/total thiol ratio also showed borderline significance (*p* = 0.050) in this subgroup analysis. Given the small number of participants in each subgroup, these findings should be interpreted as preliminary exploratory patterns. Nevertheless, they may suggest that a higher pro-inflammatory dietary load, in addition to the basal metabolic stress associated with GSD, is linked to a less favorable cellular redox profile (8, 16). This observed biological trend, when combined with the fact that diet-related cellular redox changes were not reflected by classical markers such as TAS and TOS, supports the notion that the thiol/disulfide balance may be a more sensitive parameter in specific and complex metabolic profiles (14). It is also possible that patients with lower c-DII scores made dietary choices that were more consistent with individualized recommendations; however, because adherence was not formally assessed, this interpretation remains speculative.

One of the notable findings of our study is the observed association between c-DII and higher cellular disulfide levels. This association suggests that dietary inflammatory potential may be related to redox imbalance in pediatric patients with GSD. However, given the cross-sectional design and small sample size, this finding should be interpreted as hypothesis-generating and requires confirmation in larger prospective cohorts. In the literature, numerous studies have demonstrated that DII and c-DII scores are associated with inflammatory markers and oxidative stress in various chronic and metabolic conditions. Large-scale studies have revealed that high pro-inflammatory diet scores significantly predict not only classic inflammatory markers such as CRP but also indicators of cellular oxidative stress (bilirubin, albumin, etc.) (8, 36). These findings provide broader biological support for the observed association between dietary inflammatory potential and redox status; however, they do not establish the clinical predictive utility of c-DII in pediatric GSD. Indeed, it is known that anti-inflammatory dietary approaches in chronic metabolic diseases help maintain redox balance by increasing cellular antioxidant capacity (37, 38). In this context, reducing the dietary inflammatory potential—not just controlling its energy and macronutrient content—may represent a relevant medical nutrition strategy that warrants further investigation in GSD (39). In conclusion, these findings suggest that c-DII may be a useful dietary indicator associated with cellular redox status in pediatric patients with GSD; however, this exploratory association requires confirmation in larger prospective studies.

In conclusion, this exploratory study suggests that the dietary pro-inflammatory potential observed among patients with GSDI and GSDIII receiving medical nutritional therapy may be associated with cellular oxidative damage (increased disulfide levels) and subtype-specific clinical markers (hyperuricemia and elevated CK levels). Our findings suggest that a pro-inflammatory dietary pattern may represent an additional factor associated with the cellular stress and metabolic/myopathic processes inherent to the disease. Furthermore, it was observed that the thiol/disulfide balance may be a more sensitive parameter for detecting these diet-related cellular redox changes compared to classical TAS/TOS markers. The observed associations suggest that c-DII may warrant further investigation as a dietary indicator related to cellular redox status and a potential adjunctive assessment marker in pediatric GSD. However, to establish the routine clinical utility of the c-DII score in patient monitoring, larger, longitudinal, and multicenter studies are warranted.

In this context, in the long-term management of GSD cases, there is a need for new nutritional strategies that will improve the diet's anti-inflammatory capacity within medical limits, in addition to the primary goal of preventing hypoglycemia. These findings should not be interpreted as criticism of medical nutrition therapy, which remains essential for maintaining metabolic stability and preventing life-threatening complications. Rather, they highlight the complementary role of individualized dietetic counseling in assessing actual dietary intake and improving dietary quality without compromising metabolic safety. Depending on the GSD subtype and individual tolerance, this may include increasing the variety of permitted antioxidant- and fiber-containing foods and selecting nutritionally favorable carbohydrate and fat sources where clinically appropriate.

There are certain limitations that should be taken into account when evaluating the results of our study. First, the cross-sectional and single-center nature of our study precludes causal inferences. Second, although GSD is a rare group of metabolic disorders, the small overall sample size and limited subgroups (*n* = 8 per subtype) reduce statistical precision and limit the generalizability of the findings. In addition, the median-based c-DII subgroup analysis included only eight participants per group and was therefore underpowered for definitive subgroup inference. Although the observed differences may be biologically informative, they should be regarded as exploratory and hypothesis-generating. Third, although measures were taken to improve portion-size estimation, the c-DII was calculated from retrospective dietary reports provided by patients and/or their families and may therefore remain susceptible to recall and reporting bias. Because adherence was not a prespecified outcome and was not assessed using standardized criteria, the observed dietary intake could not be formally compared with each patient's prescribed medical nutrition plan. Finally, to validate the generalizability of our findings and further elucidate the relationship between dietary inflammatory potential, cellular redox imbalance, and disease-related factors across GSD subtypes, there is a need for larger, multicenter, and long-term prospective cohort studies.

## Data Availability

The original contributions presented in the study are included in the article; further inquiries can be directed to the corresponding author.
